# Radiation dose management systems—requirements and recommendations for users from the ESR EuroSafe Imaging initiative

**DOI:** 10.1007/s00330-020-07290-x

**Published:** 2020-09-21

**Authors:** Reinhard W. Loose, Eliseo Vano, Peter Mildenberger, Virginia Tsapaki, Davide Caramella, Johan Sjöberg, Graciano Paulo, Alberto Torresin, Sebastian Schindera, Guy Frija, John Damilakis, on behalf of the European Society of Radiology (ESR)

**Affiliations:** 1 Institute of Medical Physics, Hospital Nuremberg, Prof.-Ernst-Nathan-Str. 1, 90419 Nuremberg, Germany; 2 Institute of Radiology, University Hospital, Erlangen, Germany; 3 Radiology (Medical Physics), San Carlos University Hospital, Madrid, Spain; 4 Department of Radiology, University Medical Center Mainz, Mainz, Germany; 5 Medical Physics, Konstantopoulio General Hospital, Nea Ionia, Greece; 6 Department of Radiology, University of Pisa, Pisa, Italy; 7 Medical Radiation Physics and Nuclear Imaging, Karolinska Universitetssjukhuset, Stockholm, Sweden; 8 IPC - Escola Superior de Tecnologia da Saúde de Coimbra, Coimbra, Portugal; 9 Medical Physics Department, Ospedale Niguarda, Milan, Italy; 10 Institute of Radiology, Cantonal Hospital, Aarau, Switzerland; 11 Descartes University Paris, Paris, France; 12 University of Crete Heraklion, Heraklion, Greece; 13 European Society of Radiology, Vienna, Austria

**Keywords:** Dose management systems, Radiation protection, Optimisation, Quality assurance

## Abstract

**Abstract:**

The European Directive 2013/59/Euratom requires member states of the European Union to ensure justification and optimisation of radiological procedures and store information on patient exposure for analysis and quality assurance. The EuroSafe Imaging campaign of the European Society of Radiology created a working group (WG) on “Dose Management” with the aim to provide European recommendations on the implementation of dose management systems (DMS) in clinical practice. The WG follows Action 4: “Promote dose management systems to establish local, national, and European diagnostic reference levels (DRL)” of the EuroSafe Imaging Call for Action 2018. DMS are designed for medical practitioners, radiographers, medical physics experts (MPE) and other health professionals involved in imaging to support their tasks and duties of radiation protection in accordance with local and national requirements. The WG analysed requirements and critical points when installing a DMS and classified the individual functions at different performance levels.

**Key Points:**

• *DMS are very helpful software tools for monitoring patient exposure, optimisation, compliance with DRLs and quality assurance.*

• *DMS can help to fulfil dosimetric aspects of the European Directive 2013/59/Euratom.*

• *The EuroSafe WG analyses DMS requirements and gives recommendations for users.*

## Introduction

The European Directive 2013/59/Euratom (EU-BSS) [1] sets out the basic safety standards for protection against the dangers arising from exposure to ionising radiation. The directive must be implemented into national law by all European Union member states and is important for patients as it ensures adequate radiation protection and optimisation for anyone undergoing medical imaging procedures. Implementation of the directive may be complicated in some aspects, because it only sets out very general and often unspecific standards. It is then up to individual countries to interpret these when implementing the directive into national law. In recent years, different interpretations and questions have been raised by professionals as well as by scientific and professional societies, which are relevant to harmonise the criteria for implementation of the EU-BSS in the European Union.

The EuroSafe Imaging campaign is an initiative of the European Society of Radiology (ESR). One focus of EuroSafe Imaging is helping practitioners in EU member states in the implementation process of the European Directive 2013/59/Euratom (EU-BSS) [1, 2]. This work is done by different working groups established as part of EuroSafe Imaging.

In 2019, EuroSafe Imaging launched a new working group (WG) on “Dose Management” with the aim to provide European recommendations on the implementation of dose management in clinical practice. The WG follows Action 4: “Promote dose management systems to establish local, national, and European diagnostic reference levels (DRL)” of the EuroSafe Imaging Call for Action 2018 [3].

Occupational exposures are not the focus of this WG, but may be included later.

Dose management systems (DMS) are recommended but not mandatory for X-ray equipment users, to comply with new radiation protection requirements like the EU-BSS. DMS are designed for medical practitioners, radiographers, medical physics experts (MPE) and other health professionals involved in imaging to support their tasks and duties of radiation protection in accordance with local and national requirements. In particular, the requirement according to the ALARA principle is to perform X-ray examinations with the aim to achieve a minimum dose level but maintaining a sufficient image quality or diagnostic accuracy, for the clinical indication. Clinical radiation protection includes some specific tasks which can by supported by DMS:

- Collecting dosimetric data to establish local or national DRLs or typical (median) dose values [4]
- Checking compliance with DRLs [5]
- Prevention, detection and helping in reporting of unintended exposures [6]
- Optimisation of patient exposure, especially in the field of computed tomography (CT) and interventional radiology (IR)
- Structured consolidation of dose documentation, reporting and tracking
- Notification if local or national alert levels are exceeded
- Local, regional or national benchmarking of patient exposure for modalities and procedures [7]

This results in direct requirements for basic functionalities of a DMS to achieve the above goals. The following functionalities should be mandatory.

In any case, automatic transmission of exposure data should be possible for computed tomography (CT) and interventional radiology (IR), as the data due to their higher radiation dose levels must be analysed especially for these modalities. The EU-BSS puts a special focus on exposures from CT and IR modalities (Art. 60 3. (d, e) and Art. 61 1.). For the implementation of all tasks, a complete connection of all devices is necessary even if central connection to the PACS is preferable. However, this can be limited to some extent when using computed radiography (CR) systems, as not all relevant data are automatically attached to the image data. The transfer of the data into the database of a DMS can be done in different ways but should be done automatically. Direct network connections to the imaging modalities, the image archiving and communication system (PACS) and the radiological information system (RIS) are recommended, depending on the systems used.

It must be ensured that each modality dose descriptor and the respective examination parameters used, including information on the device, are transferred to the database of the DMS in order to enable targeted evaluation. Manually recorded dose descriptors are always problematic, since this method has the potential to store incorrect or not useful values in the database. This is a situation occurring frequently in practice leading to incorrect values in a DMS. An IT infrastructure in which all dosimetric data are automatically linked to the image or automatically transferred to the RIS or PACS is the better solution. It is considered essential to organise a systematic quality control procedure to ensure that the transferred data are correct.

In CT scanners and interventional workplaces, all exposure parameters are usually stored together with the image information either in the image DICOM headers or RDSR dose report. Here, however, the problem is more often that essential dose information in older systems is stored as a secondary capture or screenshot. Therefore, it is also a requirement that advanced DMS are able to extract the texts and numbers from this information. This requires an efficient OCR system for text recognition adapted to the respective device in order to correctly assign the information. Older generation systems may also require data transfer procedures to be activated using Modality Performed Procedure Step (MPPS).

DMS must be able to correctly assign information sent in the form of the Radiation Dose Structured Report (RDSR) and dosimetric data extracted from DICOM image headers to the corresponding examinations, series or images.

The analysis of dosimetric data inserted in the database must support the listed tasks:

- To calculate median doses for individual types of examinations and devices in order to check compliance with the local, regional or national DRLs.
- If unintended exposures according to the EU-BSS occur, to alert automatically the radiation protection officer and the MPE so that it can be determined whether a reportable event has occurred.
- To obtain information on the temporal development of the median dose for individual types of examinations in order to be able to quickly determine the success of optimisation measures.
- If required, to be able to display exposure parameters, statistically processed, in order to enable optimisation.

In addition to the basic functionalities listed above, other functionalities are helpful, but not mandatory. For each dose entry, a link to the corresponding image or series of an individual examination should be provided so that the images can be accessed quickly in order to visually assess the patient’s dimensions and the image quality. Some manufacturers use Size Specific Dose Estimate (SSDE) in computed tomography to take patient dimensions into account. Radiographers could also assign height and weight to a patient manually before a scan. If possible, this can be done by a hospital information system (HIS) link transfer to the DMS. In interventional radiology, deterministic radiation effects to the skin are of particular relevance for large or adult patients. The air kerma-area product (*P*_ka_) and total air kerma at the patient entrance reference point (*K*_a,r_) are the most common parameters for fluoroscopic patient exposure, but do not allow a correct estimate of the skin dose. The representation of a skin dose mapping and the maximum skin dose (peak skin dose) offered by some DMS allows a better estimation of the skin dose during an individual procedure, with different tube positions and projections taken into account. A calculation of the effective dose for individual procedures is provided by some vendors but, due to high uncertainties, should be handled with care and provide information on the used approaches and uncertainties. The functionality offered by several manufacturers of DMS for calculating the cumulative effective dose of a patient is not considered necessary but may be helpful in specific cases of non-justified repeated exposures. The concept of effective dose is not intended for individual patients according to the ICRP recommendations.

## Requirements

In addition to the requirement profile, the prerequisites for the installation and operation of a DMS in a clinical environment should also be checked or created. Each DMS requires a considerable amount of work for configuration and maintenance. The database of a DMS receives new exposure data every day. A considerable amount of work is involved in the maintenance of the device configuration, the types of examination and protocols; furthermore, incoming data must be regularly checked for plausibility and completeness. Examination protocols must be kept up-to-date and, depending on the number of users of a DMS, the administrative workload should not be underestimated. Depending on the number of integrated devices, the number of protocols and the users, the complete configuration time of a DMS can take a few months. The assignment of examination protocols to corresponding DRLs and to standardised names of protocols using internationally recognised nomenclators to enable comparisons between different national and international centres must also be taken into account.

It is of vital importance to ensure that the dose descriptor units and dimensions of each modality, integrated into the DMS, are always the same, using the correct conversion formulas whenever necessary.

A MPE should be involved in the supervision of a DMS. The MPE should have the interdisciplinary background knowledge to aid the hospital to comply with legal requirements, to organise the required network structure together with IT, to determine which clinical data can be used, to check the clinical data for plausibility and to set alarm triggers for defined events or abnormal patient exposures. However, the MPE is dependent on the collaboration with physicians, radiographers and IT staff.

## Integration into the IT infrastructure

The basic tasks of a DMS include optimisation, QA, monitoring of national and local DRLs, detection of significant events or exposures and dose documentation of patients. In order to ensure this, it is essential to integrate the system into a central IT structure. This integration includes different levels of administrative data structures. Here, the level of patient data must be mentioned, to ensure that a patient has a unique ID, name, date of birth, sex, etc. in all systems in order to guarantee unique identification. Since normally all patient data are managed/changed in a super ordinated master system (HIS or RIS), the DMS must track any changes. Otherwise, the dataset in the systems will diverge and reliable dose documentation will no longer be possible. Manual maintenance of such changes is only conceivable in very small institutions and is not recommended even there. This data reconciliation is generally performed by an HL7-ADT interface, which communicates all changes of patient data and patient merges to the subsystems. Another level is the examination level. In daily routine, there may be manual entries of patient data at the modalities. This can happen for various reasons, such as an emergency examination, the failure of an IT system or impatience of the staff. A classic example is a polytrauma patient, who is often entered with unspecific patient data such as “Motorbike, accident 18:10”. Therefore, every PACS must have administration functions to assign image data to the correct patient and examination. Similarly, it must be possible to assign the dose data in the DMS accordingly. In order to detect incorrect dose data, it is necessary to transfer the order and examination data from the superordinate system to the DMS. Existing dose data can be checked against the orders. If there are no dose data, a manual correction is necessary, and the causes must be determined. Furthermore, the medical conditions and questions documented in the HIS can also be transferred via the order-entry interface. This allows a better classification of the procedures and a valid assignment to DRLs. Such an order-entry interface should be implemented using an HL7-ORM interface. As a DMS also stores patient data, compliance with national or federal data protection regulations must be ensured. This may be difficult to achieve with cloud solutions offered by some vendors.

## Device interfaces and data flow

**Fig. 1:**
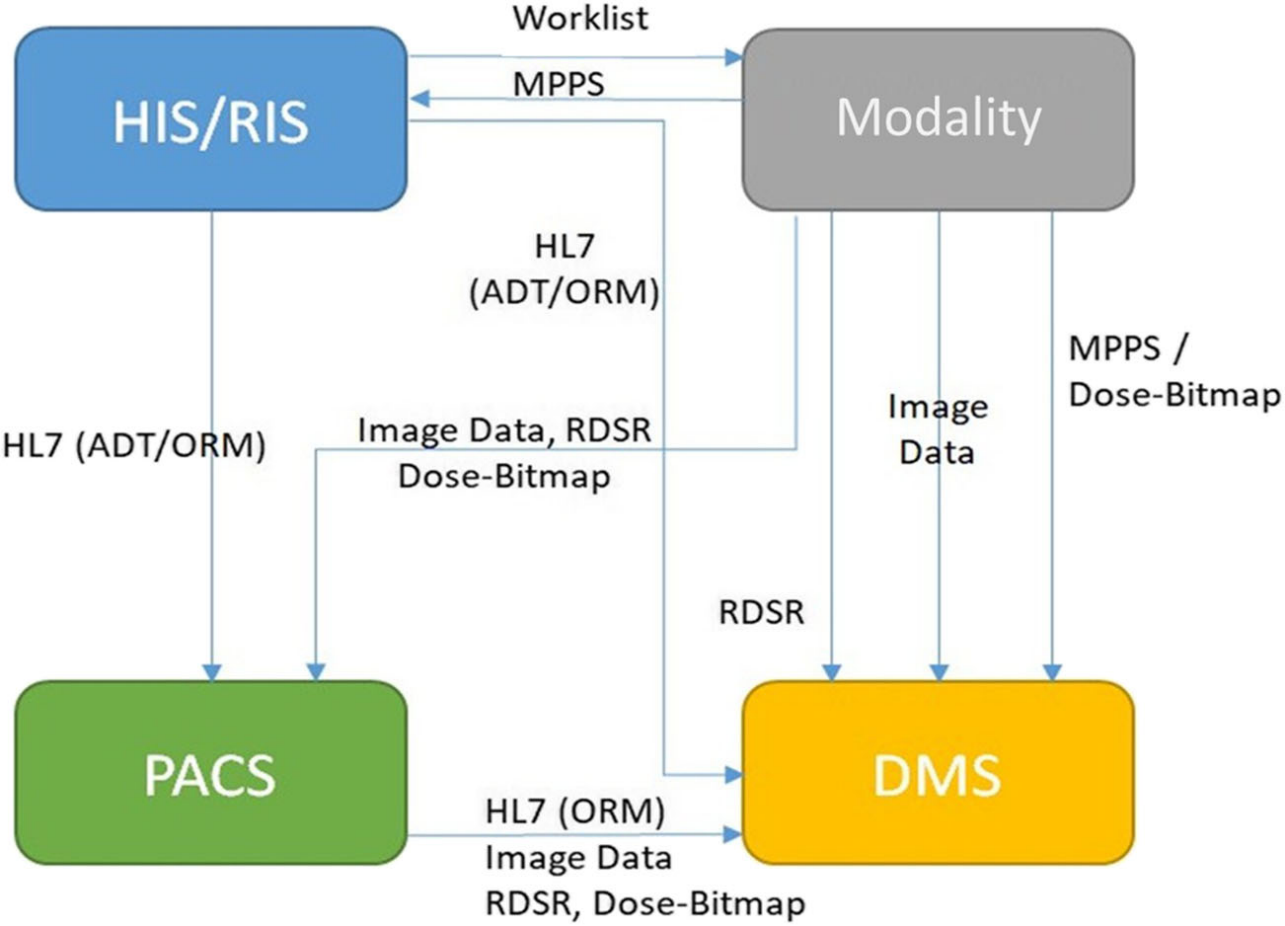
Workflow of dose management (DMS) in an environment of HIS, RIS, PACS and modalities (B. Renger, Munich, 2019)

It was agreed in both WGs (“dose management” and “dosimetry for imaging in clinical practice”) that dosimetric input data for DMS should be only the dose descriptors provided by the different modalities, properly validated by a MPE in close cooperation with the physician and the radiographer. Depending on the workflow, these data may either come directly from the modalities or with only one source via the PACS. Here, the WG will follow recommendations of the WG “dosimetry for imaging in clinical practice”. In principle, all connections are possible and can be realised. The preferred method depends on the local infrastructure and cannot be answered generally. A mixed installation is not advisable, since troubleshooting can be very complicated and additional error possibilities arise. Besides the question of data flow (directly or via a subsystem) (Fig. 1), the transfer mode must also be determined. The following transfer of dosimetric data is available:

- DICOM Radiation Dose Structured Report (RDSR)
- DICOM Modality Performed Procedure Step (MPPS)
- DICOM header of image data
- DICOM images with bitmap dose reports (OCR recognition)

### RDSR

If available, RDSR is the preferred transfer mode. Both cumulative and series-related exposure data are transferred and stored in a structured way. In addition, an RDSR object can also be stored in the PACS and thus archived in a legally compliant manner. In case of fluoroscopic procedures, RDSR transfers not only the image/series-related doses but also the dose of fluoroscopy events without images. In addition, for each fluoroscopy scene parameters like field of view, tube position, pulse rate, and scene length are transferred. This allows calculation of dose maps and peak skin dose. A drawback for CT is that the RDSR dose parameter CTDI_vol_ is the dose average over all slices of a series. Hence, the effect of dose modulation along the z-axis is lost and only can be retrieved by analysing the header of each single slice.

### MPPS

The use of MPPS data is an older concept. MPPS is intended for workflow control and has been withdrawn from the DICOM standard. Many modalities send cumulative and partly series-related dose data with the completion of the examination at the device.

### Image data

Using the DICOM metadata associated with the image data can be a good alternative or addition for transfer of dosimetric data. In most cases, many other examination parameters that are not available in the RDSR can be extracted. However, this is inevitably associated with a high network load and system load if the image pixel data are not stripped from the headers. Some DMS cannot distinguish between secondary reconstructed images and the primary acquisition (e.g. in CT). Therefore, examinations with one primary acquisition and several secondary reconstructions with multiple dose entries can be registered in a DMS. When using image data, this should be checked carefully.

### DICOM images with bitmap dose reports

Many CT and fluoroscopic modalities store dose reports in the form of DICOM bitmap images when closing the examination. These are primarily intended as readable documents, not for data transfer to a DMS system. Depending on the manufacturer and software version, relevant information such as the reference phantom may also be missing. However, many DMS offer data extraction from these images by means of OCR recognition. Anyway, errors in an OCR process cannot be neglected. Similar to MPPS data, this is an older concept and should only be used for modalities that do not support RDSR.

## Classification of procedures and assigning to DRLs

DRLs are defined differently for different types of modalities and procedures. Depending on the type of modality and national regulations, they can be defined at image, series or examination level. Therefore, a DMS must be able to classify individual exposures, series and complete examinations and to monitor them with the respective DRL. The sole use of the protocol names or the requested procedure name has not proven to be sufficient. In practice, different procedures are performed with the same protocol name and vice versa. Also, different protocols are linked together in one procedure or only individual parts of the respective protocols are used. This places high demands on DMS, especially for classification at series level. A possible solution to this problem is the rule-based linking of different parameters, which is already offered by some DMS manufacturers. The parameters used in rule-based linking are different for each type of modality.

Standardised names of the protocols using internationally recognised nomenclature should be included. The RadLex® glossary is one example.

### CT—exposure level

In order to correctly classify CT exposures and compare them with the correct DRLs, it should be possible to use the following parameters:

- Procedure name
- Series description
- Number of phases/series
- Protocol name
- Body region

#### Reference phantom size

- Type of acquisition
- Scan length

### Radiography and mammography—exposure level

In order to clearly classify radiographic exposures and compare them with the correct DRLs, it should be possible to use the following parameters:

- Procedure name
- Protocol name
- Body region
- Projection
- Patient orientation

### Fluoroscopy and intervention—examination level

Classification at procedure level is difficult for fluoroscopic and interventional examinations. On the one hand, many different procedures are performed with the same protocol; on the other hand, there are many medical variations for a single procedure name. Medical societies should be involved in defining the most frequent clinical procedures (ICRP uses the term “clinical tasks”) and promote the evaluation of complexity level to be implemented in the DRLs [8].

The European Commission launched a “European Study on Clinical Diagnostic Reference Levels for X-ray Medical Imaging” (EUCLID) in 2017. EUCLID was carried out under the coordination of the ESR. The main task was to collect data from European hospitals and determine up-to-date CT and IR DRLs based on clinical tasks. It was found that DMS can facilitate data collection and help in establishing clinical DRLs.

## Dose monitoring and alert thresholds

DMS should continuously monitor the dosimetric parameters to ensure that DRLs are not consistently exceeded and alert thresholds are monitored. In addition to these basic requirements, it must be possible to use local DRLs and alert levels as test criteria for radiation protection optimisation. The use of local DRLs and alert levels is recommended as for many procedures national DRLs do not exist.

On the other hand, with newer devices, the dose can be substantially below national DRLs, particularly if they were not updated for a longer time, and it should be possible to use other values for checking the level of radiation exposure. If the DMS detects abnormal values, the MPE or radiation protection officer should be informed; this could be, e.g. in the form of an email or message on the computer used. A control as to whether violations have occurred should be carried out frequently, optimally every working day. For this, it is helpful that the DMS has an appropriate information tool that can quickly call up this information. Depending on the form of the overrun, immediate measures may be necessary and must be implemented. Since the exposure parameters used are also stored in the DMS, this information can also be used to monitor changes in examination parameters. With changes, e.g. the tube voltage, the DMS can inform the MPE or the radiation protection officer about this, so that he can check the problem. This is necessary because, e.g. in the case of software updates on the part of the manufacturer or by user intervention, undesirable changes to the examination protocols and parameters can take place. This possibility of monitoring and thus optimising radiation protection is possible for the first time without much time and should be used accordingly. In addition to monitoring changes, it is also possible to compare the device parameters used for identical types of examination and can be used to better optimise and standardise the examination techniques and the exposure to radiation that they involve. Statistics on the examination protocols used make it possible to identify any duplicate protocols that may occur for individual examination types and to summarise and standardise them.

## Statistical evaluation

Statistical evaluations of the collected dosimetric data in a DMS are the basis for analysis, optimisation and clinical audit. The sole detection of examinations with DRL exceedances is not sufficient for these tasks. The distribution of the dose data is much more important. For all statistical evaluations, it must be possible to anonymise and export the data.

Processed tables should identify:

- Unique modality identification, e.g. device type, manufacturer, model, internal device name, and serial number.
- Number of examinations performed per modality and selected period with listing of protocol name, procedure description, series description and body region.
- List of the individual exposure events with all available protocol parameters. In addition to the information listed above, other dose-relevant variables can also be taken into account, such as exposure index (EXI), total *P*_ka_, DLP, CTDI_vol_, SSDE, pulse rate, filter type and tube angulation.
- Dose values (median, mean, minimum, maximum, standard deviation, number of procedures evaluated) per modality, procedure and protocol for a specified period.
- List of procedures exceeding national or local DRLs per modality, procedure and protocol in a defined time period.

It should be possible to create representative graphs from the tabulated data.

## Performance levels of DMS

One topic initially selected by the WG was the classification of different DMS performance levels. A number of software systems for dose management have come onto the market in recent years. They offer different solutions such as locally installed software, cloud solutions, commercial systems or pay per use solutions. Depending on the solution and manufacturer, this results in a price range from a few thousand to over 100,000 Euros, depending on the complexity and number of analysed exams. The choice of a specific solution depends on the size of an institution, the number of modalities to be connected, the number of examinations and the respective requirements for a DMS. One topic initially selected by the ESR WG “Dose Management” at a local meeting at ECR 2019 and in an online conference in October 2019 was the proposal to classify DMS solutions into different performance levels. Initially, the WG proposed three levels:

- Basic requirements
- Standard requirements
- High-level solutions

### DMS basic requirements

- These systems should use physical device–related DICOM dose parameters and work with DX (CR with DAP interface), MG, CT, RF, XA.
- Systems must get acquire and store dosimetric parameters either from DICOM headers of all images (image/pixel data may be skipped) or from DICOM RDSR reports.
- Store dose data in a database, e.g. with SQL query.
- Local procedure names should be translated into standardised procedure names (e.g. national name lists or RadLex playbook or equivalent) to enable comparison and benchmarking of referrals and procedures in different institutions or countries [9].
- Set alert trigger levels (local and national).
- Export dose data for optimisation, QA, reporting (e.g. to national authority) and post processing

### DMS standard requirements (all above plus)

- Display dose charts as timeline for selected modalities and procedures
- Automatic reports to responsible professionals (radiologists, radiographers, MPE)
- Send automatic alerts to above professionals on local/national events
- Input of dosimetric data via MPPS
- OCR recognition of bitmap dose reports (optional manual data entry)
- Link from any dose parameter to open the corresponding image

### DMS high-level solutions (all above plus)

- Report modality load (day, time, procedure)
- Optionally include contrast media data
- Calculation of organ doses (with high uncertainties)
- Calculation of effective dose for individual patients (not recommended by ICRP due to large uncertainties)
- Store and display cumulative patient dose [10]
- Include occupational doses (optional)

## Summary

ESR encourages users of radiological modalities to use DMS wherever possible and appropriate. DMS can be applied as quality control for monitoring patient exposure, assistance of protocol optimisation, checking compliance with national and local DRLs, providing exposure data to authorities and detecting unintended exposures.

It was agreed that there is a need for DMS which can be tailored to the size and workload of a clinic/institution. A large maximum care hospital has other requirements than a small institution with one practitioner and one or two X-ray modalities. Large-scale DMS require a significant staff workload and expertise, especially by MPEs.

DMS should be configurable to include national requirements, like DRLs and DRLs based on clinical indication, or other trigger levels. It must be clearly defined if exposure data are linked on image, series or procedure level. The integration of interventional procedures beyond the simple storage of air kerma-area products (*P*_ka_) is complex, as calculation of skin doses and skin dose mapping requires RDSR datasets with field size, tube and C-arm positions for all fluoroscopic steps of a procedure. Challenges are criteria for detection and reporting of unintended exposures as there is a wide range of implementations of the EU-BSS into national law like absolute doses, multiples of DRLs, effective dose or just plain text.

In summary, there are a number of important requirements that must be met and that determine the success or failure of the introduction of a DMS.

- A network connection with DICOM standard of all relevant modalities either to a PACS or directly to a DMS system. In any case, CT scanners and fluoroscopic modalities for interventional procedures must be integrated.
- The transmitted dosimetric data should only be physical dose parameters such as air kerma-area product (*P*_ka_), air kerma at the patient entrance reference point (*K*_a,r_), average glandular dose (AGD), volume computed tomography dose index (CTDI_vol_) and dose length product (DLP). If calculated organ or effective doses are provided, the uncertainties should always be considered.
- The preferred standard for transmitting dosimetric parameters for all modalities and procedures is Radiation Dose Structured Report (RDSR). In case of missing RDSR for mammography or radiography examinations, the exposure data can also be retrieved from the DICOM headers. The use of bitmaps with subsequent OCR analysis, Modality Performed Procedure Step (MPPS) or manual recording is outdated and no longer recommended.
- A DMS must provide translation tables or other software tools to map local procedure or protocol names into a standardised nomenclature. This is necessary for comparison with national or local DRLs, other trigger levels and for benchmarking different modalities or institutions. Without these translation tables, the data may be restricted to procedures of one institution and one modality.
- A DMS must inform radiologists, MPEs and radiographers about relevant events at different notification levels and provide statistically processed data for quality assurance, optimisation and communication with national authorities. In case of unintended exposures according to the EU-BSS, automatic alerts should inform the responsible staff members.
- During installation and subsequent operation of a DMS, the inclusion of an MPE is strongly recommended, especially in larger institutions or complex installations.

The effort involved in meeting all these requirements should not be underestimated when planning, installing and operating a DMS.

## Abbreviations

ADT: Admission, discharge and transfer
ALARA: As low as reasonably achievable
CR: Computed radiography
CT: Computed tomography
DICOM: Digital Imaging and Communication in Medicine
DMS: Dose management system
DRL: Diagnostic reference level
DX: Digital radiography
HIS: Hospital information system
HL7: Health level 7
*K*_a,r_: Air kerma at the patient entrance reference point (ICRP notation)
MG: Mammography
MPE: Medical physics expert
MPPS: Modality Performed Procedure Step
OCR: Optical character recognition
ORM: Order message
PACS: Picture archiving and communication system
*P*_ka_: Air kerma-area product (ICRU notation)
RDSR: Radiation Dose Structured Report
RF: Radio fluoroscopy
RIS: Radiology information system
SSDE: Size Specific Dose Estimate
XA: X-ray angiography
